# Generation of animals allowing the conditional inactivation of the *Pax4* gene

**DOI:** 10.1007/s11248-012-9624-0

**Published:** 2012-06-21

**Authors:** Simon Kordowich, Palle Serup, Patrick Collombat, Ahmed Mansouri

**Affiliations:** 1 Department of Molecular Cell Biology, Max-Planck Institute for Biophysical Chemistry, Am Fassberg, 37077 Göttingen, Germany; 2 Department of Developmental Biology, Hagedorn Research Institute, Niels Steensensvej 6, 2820 Gentofte, Denmark; 3 Present Address: The Danish Stem Cell Center (DanStem), Copenhagen, Denmark; 4 Diabetes Genetics Team, Inserm, U1091, 06108 Nice, France; 5 Université de Nice Sophia-Antipolis, 06108 Nice, France; 6 Department of Clinical Neurophysiology, University of Göttingen, Robert-Koch Strasse 40, 37075 Göttingen, Germany

**Keywords:** Beta-cells, Pancreas, Insulin, Knockout, Floxed allele, Diabetes

## Abstract

Pax4 belongs to the paired-box family of transcription factors. The analysis of loss- and gain-of-function mutant animals revealed that this factor plays a crucial role in the endocrine pancreas. Indeed, Pax4 is required for the genesis of insulin-producing beta-cells. Remarkably, the sole misexpression of *Pax4* in glucagon-expressing cells is able to induce their regeneration, endow these with beta-cell features, and thereby counter chemically induced diabetes. However, the function of *Pax4* in adult endocrine cells remains unclear. Herein, we report the generation of *Pax4* conditional knockout mice that will allow the analysis of Pax4 function in mature beta-cells, as well as in the adult central nervous system.

## Introduction

Pax4 is a member of the paired-box-containing family of transcription factors (Mansouri et al. 1996) that has been shown to play a crucial role in the endocrine pancreas (Sosa-Pineda et al. 1997). During pancreas morphogenesis, *Pax4* is first detected in the pancreatic epithelium and is later found confined to developing insulin-producing beta-cells, where it is down-regulated perinatally. Cell-lineage studies revealed an expression of *Pax4* in all endocrine precursors (Greenwood et al. 2007). Importantly, the analysis of the pancreas of *Pax4*-deficient animals demonstrated that this factor is an important player in the processes underlying beta-cell genesis (Sosa-Pineda et al. 1997). Indeed, in the absence of *Pax4* gene activity, while mutant islets display normal morphology and cell counts, a dramatic beta- and delta-cell loss and a proportional increase in the glucagon-producing alpha-cell content are evident (Sosa-Pineda et al. 1997). Further studies have evidenced that, during endocrine cell genesis, *Pax4* is involved in the endocrine cell subtype specification. During this process, Pax4 and Arx undergo a reciprocal cross-inhibitory interaction to endow endocrine progenitors with beta- and alpha-cell destiny, respectively (Collombat et al. 2003; Collombat et al. 2006). Arx is a homeobox-containing transcription factor that is required for the formation of glucagon-producing alpha-cells (Collombat et al. 2003). Additionally, compound mutants for *Pax4* and *Arx* suffer from a loss of beta- and alpha-cells while the delta-cell content is augmented, revealing that Pax4 is not required for delta-cell genesis (Collombat et al. 2005). Remarkably, the forced expression of *Pax4* in glucagon^+^ cells in vivo was found to induce their regeneration and subsequent conversion into functional beta-like cells that can counter chemically-induced diabetes (Collombat et al. 2009). Hence, Pax4 appears both necessary and sufficient to promote the beta-cell fate/phenotype. However, despite such important roles, the function of this factor in adult beta-cells has hitherto not been addressed. Studies using human and rat islets suggested that it might play an important role in beta-cell proliferation and survival in the adult pancreas (Brun et al. 2004). In addition, the overexpression of *Pax4* in adult beta-cells appears to protect insulin-producing cells from apoptosis (Hu He et al. 2011). We therefore used an embryonic stem cell-mediated gene targeting approach to generate *Pax4* conditional knockout mice.

## Materials and methods

### Generation of *Pax4* conditional knockout mice

**Fig. 1:**
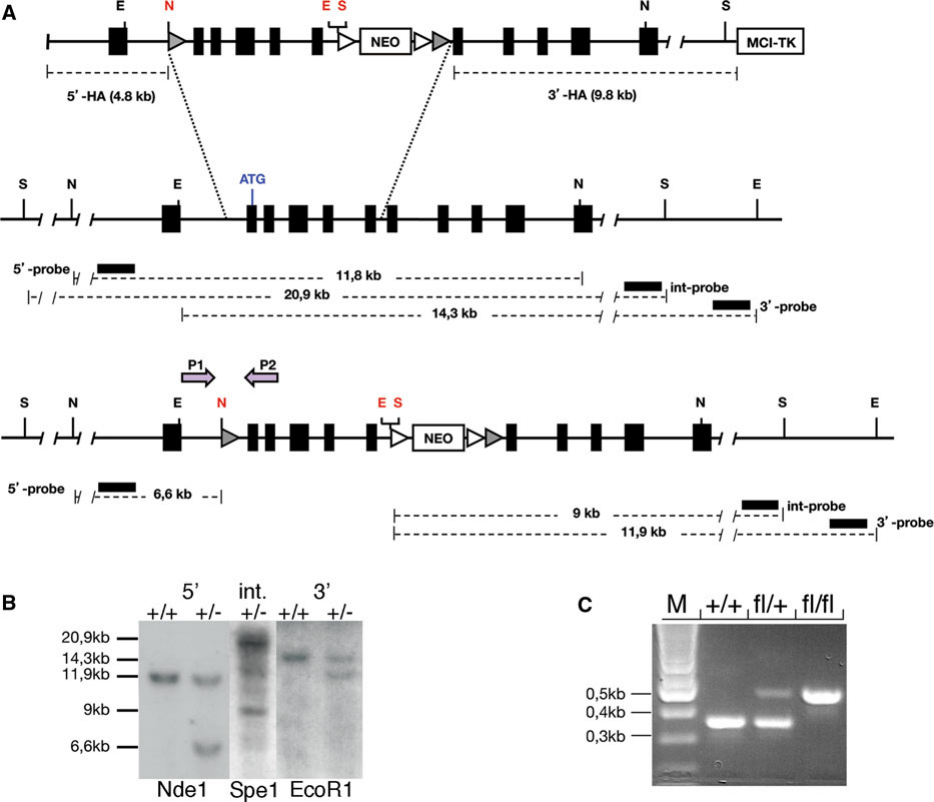
Targeting construct. The targeting construct was designed and genetic manipulations were performed using recombineering techniques (Copeland et al. 2001). Briefly, a LoxP site was introduced within the first intron and the neomycin resistance cassette carrying the second LoxP site and flanked by Frt sites within the sixth intron. 129 Bac DNA spanning the mouse *Pax4* locus was used to isolate the sequences required for the cloning of the targeting construct (**A**). For negative selection, a MCI-TK cassette was added at the 3′end to allow selection with gancyclivir (Mansour et al. 1988). The size of the 5′and 3′ homology arms (HA) is indicated. Internal and external probes as well as the location of primers for PCR genotyping are also shown.* Black boxes* indicate exons.* White arrowheads* represent FRT-sites and* dark arrowheads* LoxP-sites. **B** Genomic Southern-blot using 5′ and 3′ external, as well as internal probe; N: Nde1; S: Spe1; E: EcoRI, genotype is indicated: +/+ wild type; ± heterozygous for the floxed allele. **C** Genotyping by PCR; M: molecular weight marker; +/+ wild type; fl/+ heterozygous and fl/fl: homozygous

Using the recombineering approach (http://recombineering.ncifcrf.gov/Plasmid.asp), a *Pax4* targeting construct was generated. Specifically, one LoxP site was inserted within the first intron, and a neomycin resistance cassette was introduced within the sixth intron (Fig. 1). The neomycin resistance coding sequences are under the control of the PGK promoter. This cassette is flanked by FRT sites and followed by one LoxP-site (Fig. 1A). The thymidine kinase gene, used for negative selection, was inserted at the 3′end of the construct. The targeting construct was electroporated in MPI II ES cells and targeted clones were identified by Southern blot (Fig. 1B). Germ line chimeras were generated by morulae aggregation. Two independent ES cell lines gave germ line transmission. Genotyping reactions were performed using PCR (Primer pair 1: 5′-AGCTCCAGTGAGTTTAGAAACTGCTAGGAGGTG-3′ and 5′-CTTCCTCAAGTGGGCAACTTGACATCTGGCCCA-3′—WT allele: 358 bp; CKO allele: 466 bp; Primer pair 2: 5′-GTCTTGAGACTTATGGGTGAAGGCTGACAGGGTCC-3′ and 5′-CAGTATACATTCGTCTCCTTTACAGACCCTCACAC-3′—WT allele: 304 bp; P4CKO Allele: 417 bp). Animal care and experimental use were approved by the Ministry of Agriculture of Low Saxony (LAVES), Germany.

### Immunohistochemistry

Embryos were prepared at the indicated time points and pancreatic tissues were isolated and transferred for fixation in 4 % paraformaldehyde overnight. After dehydration, the tissues were embedded in paraffin and 8 μm sections were prepared. Following several washes in PBS for 5 min, sections were transferred into blocking solution consisting of PBS containing 10 % fetal calf serum for 1 h. Primary antibodies were diluted in the same blocking solution and incubated overnight at 4 °C. Following washes in PBS, the slides were treated for 1 h with the corresponding secondary antibodies diluted into PBS containing 10 % fetal calf serum. Slides were then rinsed with PBS, mounted with DAPI, and analyzed by fluorescence microscopy. Primary antibodies used: mouse anti Pax6 (Developmental Studies Hybridoma Bank, dshb@uiowa.edu 1:50), Rabbit anti Pax4 (1:500), kindly provided by Dr. Sosa-Pineda (Collombat et al. 2009); guinea pig anti insulin (1:1000, Dako), mouse anti glucagon (1:1000, abcam), rabbit anti somatostatin (1:600, Dako), rabbit anti ghrelin (1:1000, Millipore), goat anti ghrelin (1:50, Santa Cruz). The secondary antibodies (1/1000, Molecular Probes) used : 594-alexa anti-mouse, 488-alexa anti-mouse, 594-alexa anti-rabbit, 488-alexa anti-rabbit, 594-alexa anti-guinea pig, and 488-alexa anti-guinea pig, 594-alexa anti-goat, and 488-alexa anti-goat.

## Results and discussion

A targeting construct was designed and recombineering techniques used to introduce one LoxP site within the first intron and a second LoxP site within the sixth intron of the *Pax4* gene. As depicted in Fig. 1A, the second LoxP site is integrated into the 3′end of the neomycin resistance gene, which is flanked by FRT sites.

The knockout construct was electroporated into MPI II ES cells and 2-targeted clones were used to generate chimeric animals by morulae aggregation. Mice homozygous for the floxed *Pax4* allele were found normal and fertile. This was true for two mouse lines derived from two independent targeting events. Targeting events were confirmed by Southern blot using 5′and 3′external probes (Fig. 1B).

Following germ line transmission, the neomycin cassette was removed by crossing the generated *Pax4* animals carrying the floxed allele with transgenic mice ubiquitously expressing the Flp recombinase (Dymecki 1996). Homozygous mice derived from two independent *Pax4*-targeted ES clones for the floxed allele were also normal and fertile.

**Fig. 2:**
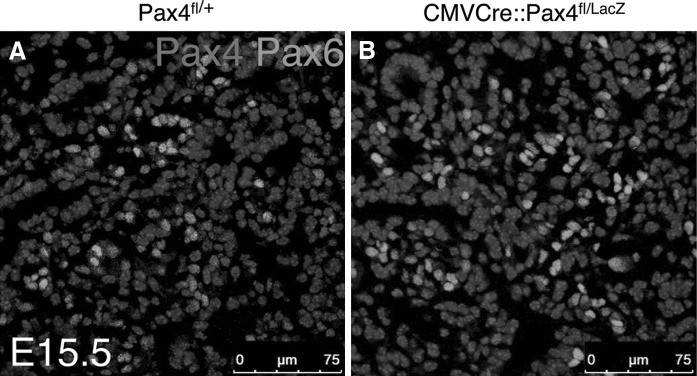
Inactivation of *Pax4* in animals harboring a *Pax4* floxed allele and CMV-Cre transgene. Section of E15.5 pancreas derived from *Pax4* ^fl/+^ (**A**) and CMV-Cre::*Pax4* ^fl/LacZ^ (**B**) mice stained for Pax4 (*red*) and Pax6 (*green*). In CMVCre::Pax4 ^fl/LacZ^ mutants, the expression of Pax4 is clearly abolished (**B**)

To determine whether *Pax4* could efficiently be inactivated, we subsequently crossed the floxed allele-carrying animals with a transgenic mouse line expressing the Cre recombinase under the control of the ubiquitous CMV (Cytomegalovirus) promoter. This was achieved by crossing previously derived *Pax4* ^+/−^ heterozygous mice in which the beta-galactosidase coding sequence had been inserted into the *Pax4* locus (Sosa-Pineda et al. 1997), with CMV-Cre animals, to generate CMV-Cre::Pax4^+/−^ double transgenic mice. These were further mated with homozygous Pax4 ^fl/fl^ animals. Some of the offspring died at birth or few days *postpartum*. Those animals were genotyped as CMV-Cre::Pax4 ^fl/LacZ^. Using immunohistochemistry on pancreas sections of E15.5 embryos, we could demonstrate that in such animals, *Pax4* expression had been abolished, confirming its complete inactivation (Fig. 2B).

**Fig. 3:**
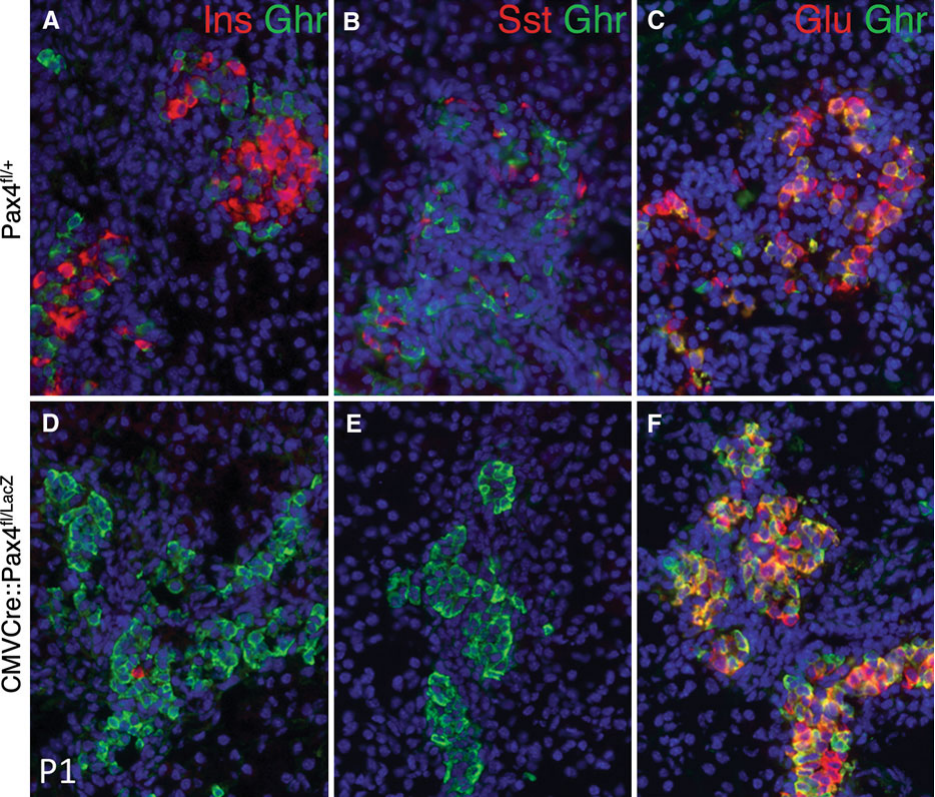
Inactivation of *Pax4* using the floxed *Pax4* allele recapitulates the phenotype reported for *Pax4* knockout (LacZ knock-in). Immunohistochemical analysis of P1 pancreata derived from Pax4 ^fl/+^ and CMV-Cre::Pax4 ^fl/LacZ^ mice for the pancreatic hormones. Mutant pancreata lack insulin- and somatostatin-producing delta-cells, while the content of alpha-cells is proportionally increased. Also, the number of ghrelin-positive cells is dramatically augmented. These alterations in hormone expression recapitulate the phenotype previously described for *Pax4* knockout mice (Sosa-Pineda 2004)

We then asked whether *Pax4* inactivation in CMV-Cre::Pax4 ^fl/LacZ^ mice could recapitulate the phenotypic alterations that were previously described for *Pax4* knockout animals using the LacZ knock-in allele (Sosa-Pineda et al. 1997). Sections from newborn (P1) pancreas derived from Pax4 ^fl/+^ or CMV-Cre::Pax4 ^fl/LacZ^ mice were subjected to immunohistochemistry to assess the expression of endocrine hormones. As shown in Fig. 3, insulin- as well as somatostatin-producing cells were found lacking whereas the alpha-cell content appeared highly augmented in the islets of these animals. Moreover, numerous glucagon-expressing cells were also found positive for the ghrelin hormone. These findings provide evidence that the observed phenotype in CMV-Cre::Pax4 ^fl/LacZ^ mice recapitulates the alterations previously described for the endocrine pancreas of *Pax4* knockout animals (Sosa-Pineda et al. 1997; Wang et al. 2008).

The reported mice carrying the *Pax4* floxed allele therefore represent a powerful tool to study the role of Pax4 in the adult pancreas, but also in the central nervous system, in tissues such as in the retina or the pineal gland (Rath et al. 2009a; Rath et al. 2009b).
